# Biogeography of amphi-adriatic *Gentianella crispata* (Gentianaceae): a northern refugium and recent trans-adriatic migration

**DOI:** 10.1080/11263504.2021.1918783

**Published:** 2021-06-01

**Authors:** Dieter Reich, Ruth Flatscher, Giuseppe Pellegrino, Karl Hülber, Johannes Wessely, Andreas Gattringer, Josef Greimler

**Affiliations:** 1 Division of Systematic and Evolutionary Botany, Department of Botany and Biodiversity Research, University of Vienna, Vienna, Austria; 2 Department of Biology, Ecology and Earth Sciences, University of Calabria, Rende, CS Italy; 3 Division of Conservation Biology, Department of Botany and Biodiversity Research, Vegetation- and Landscape Ecology, University of Vienna, Vienna, Austria

**Keywords:** Balkan Peninsula, climate change, Pleistocene refugium, refugia-within-refugia, species distribution modelling

## Abstract

The Balkan Peninsula as one of Europe’s biodiversity hotspots came into the focus of biogeographic research during the last decade, but the phylogeography of its mountain flora still remains widely unexplored. To investigate historical and future range dynamics within *Gentianella crispata*, a typical plant of mountainous grasslands sub-endemic to the Balkan Peninsula with a single known population in southern Italy, we combine molecular analyses based on cpDNA sequence and amplified fragment length polymorphism data with species distribution models. In the western Balkan, we found a distinctly separated cluster at the northern edge of the species’ distribution range. The split towards the less structured southern populations is located at the Neretva canyon, a pattern repeatedly reported before. This finding is clearly in line with the refugia-within-refugia hypothesis assuming more than one Pleistocene refugium on the Balkan Peninsula. The Italian population was found to be closely related to populations in the central to southern Dinaric mountains indicating a recent, possibly human-mediated migration event. Niche models for future climate scenarios forecast a severe loss of climatically suitable areas, especially in the southern parts of the range, pointing at deteriorating effects of climate warming for those mountainous grassland habitats in southern Europe, which harbour *G. crispata*.

**Supplementary Material:**

The online version contains supplementary material available at https://doi.org/10.1080/11263504.2021.1918783.

## Introduction

The Balkan Peninsula is a mountainous region with more than 60% of its area at elevations above 1000 m a.s.l. (Willis 1994). In contrast to the neighbouring European Alps, mountain chains at the Balkan (i.e. Balkan mountains, Dinarides, Pindus mountains, Rhodopes) did not have a continuous ice shield during the last glacial maximum (LGM) but only the highest peaks were glaciated for longer time periods (Menkovic et al. 2004; Milivojević et al. 2008). As a consequence, the Balkan Peninsula was an important refugial area for mountain organisms (Schmitt 2007). At recent times it is a major biodiversity hotspot in Europe (Kryštufek and Reed 2004) harbouring more than 6500 plants species with a high rate of endemism (Horvat et al. 1974; Stevanović et al. 2007). Regardless of this high importance, the biogeography of the region is still under-investigated compared to the other Mediterranean Peninsulas, but the number of case studies steadily increases.

Even in the absence of strong differentiating effects of large-scale glaciation on species distributions, distinct phylogeographic splits and regional refugia have repeatedly been identified in animals (e.g. Mezzasalma et al. 2018; Vucić et al. 2018) and plants (e.g. Surina et al. 2011; Kutnjak et al. 2014; Jug-Dujaković et al. 2020). This supports the refugia-within-refugia scenario (Gómez and Lunt 2007) assuming that the southern European peninsulas were not only important refugia altogether during the Pleistocene but themselves structured into several local refugia. For the Balkan Peninsula Médail and Diadema (2009) proposed four refugia along the Adriatic coast (i.e. (i) northern Istria, the (ii) Velebit and (iii) Biokovo mountains in Croatia and (iv) the coastal Montenegro mountains of Orjen and Lovčen) and four refugia in Greece (i.e. the Pindus mountains, the SW Peloponnese, Mt. Olympus and the Chalkidiki Peninsula). However, biogeographic patterns are strongly idiosyncratic and there are still too few studies to conclusively identify local refugial areas as done for the Alps (Schönswetter et al. 2005).

Besides endemism, amphi-adriatic distributions are long known as a conspicuous biogeographic pattern regarding many plant species of the Balkan Peninsula (Turrill 1929). In many cases such species have a large distribution area on the Balkans and isolated, regional to locally restricted occurrences in southern Italy like *Inula verbascifolia* (Willd.) Hausskn. or *Satureja cuneifolia* Ten. (Di Pietro and Wagensommer 2014). In addition, large distribution areas on both sides of the Adriatic Sea can frequently be found, as exemplified in *Drypis spinosa* L., *Geocaryum cynapioides* (Guss.) Engstrand [as *Huetia cynapioides* (Guss.) P.W. Ball], *Geranium versicolor* L., *Lathyrus grandiflorus* Sm., *Ranunculus brutius* Ten. or *Thymus striatus* Vahl (Di Pietro 2009). In contrast, taxa being widely distributed on the Apennine Peninsula but having only a very restricted range on the Balkan Peninsula as found in *Echinops siculus* Strobl (Conti et al. 2020) or *Potentilla rigoana* Th. Wolf (Dobeš et al. 2013) are comparatively rare.

Reasons for these distribution patterns are still contentious. A land bridge connecting the Apennine with the Balkan Peninsula (Popov et al. 2006) during the so called Messinian Salinity Crisis (MSC, 5.96 − 5.33 Ma) was considered as one explanation (Turrill 1929). However, molecular evidence for such a temporal land bridge causing amphi-adriatic distribution patterns is rare. As an example, the split between the Balkanian *Campanula poscharskyana* Degen and the Italian *C. garganica* Ten. was dated roughly at the time of the MSC (Frajman and Schneeweiss 2009). Another reason for amphi-adriatic distributions could be a low sea level or even land bridges at the end of the LGM, allowing for more recent migration (Correggiari et al. 1996) as suggested for *Knautia drymeia* Heuff. (Rešetnik et al. 2016b) or the *Euphorbia barrelieri* group (Frajman and Schönswetter 2017). In addition, long distance dispersal (LDD) events are possible in principle, but were inferred only for *Edraianthus graminifolius* (L.) A. DC. (Surina et al. 2014). Moreover, migration pathways could have followed the coast line of the Adriatic Sea as proved for the *Silene saxifraga* group (Durović et al. 2017). Finally, transportation facilitated by human activities cannot be ruled out, especially in taxa inhabiting mountainous grasslands traditionally used for pasture farming or nomadic grazing. Anthropogenic amphi-adriatic dispersal was considered for several Gastropoda (Korábek et al. 2014) or a termite species (Scicchitano et al. 2018). In plants, human-mediated LDD was reported only for a few more wide-spread neophytic taxa naturalized from cultivation like *Aesculus hippocastanum* L. or *Syringa vulgaris* L. as well as for spontaneous neophytes like *Capsella grandiflora* (Fauché & Chaub.) Boiss., *Tanacetum cinerariifolium* (Trevir.) Sch. Bip. or *Trifolium dalmaticum* Vis. (Celesti-Grapow et al. 2009). The vast majority of these migration events are assumed to have been directed from the Balkan Peninsula towards Italy (Frajman and Schönswetter 2017).

Past changes in the distributions of species are, hence, likely to have emerged from the combined effects of climatic changes and the species-specific migration history, the latter potentially supported by direct human activities. Accordingly, we can expect a significant influence of anthropogenic climate change on the future distribution of species (Radenković et al. 2017; Fassou et al. 2020). Thereby, cold-adapted taxa of mountainous to high-mountain habitats are expected to be highly vulnerable to climatic changes in the twenty-first century (Dullinger et al. 2012). Along a latitudinal gradient, climate change effects are assumed to be more pronounced the further south on the Mediterranean Peninsulas (Radenković et al. 2017), but the knowledge on future distributions of mountain plants at the Mediterranean Peninsulas in general, and at the Balkans in particular, is very limited (Kutnjak et al. 2014).

Despite the steadily growing number of biogeographic studies investigating taxa of the Mediterranean Peninsulas, knowledge on the phylogeography of Mediterranean plants of high altitude grasslands as *Gentianella crispata* (Vis.) Holub is still scarce. We use *G. crispata* to showcase the Pleistocene and postglacial range dynamics of plant species between the Balkan and Italian Peninsula. This species represents an excellent study system as it combines a strong geographic disjunction - the main range at the Balkans and a single isolated population at the Apennine - with potentially very low dispersal abilities deduced from the lack of any morphological adaptations facilitating LDD. According to age estimates for splits within the genus (Bernhard von Hagen and Kadereit 2001), *G. crispata* most likely is too young to have migrated via land bridges during MSC. This indicates strongly contrasting migration histories: either an old migration event during the late Pliocene or the Pleistocene or a more recent, maybe human mediated dispersal. A more solid knowledge of the species’ responses to past changes in climatic conditions will likely allow us to better assess *G. crispata*’s fate in the face of future anthropogenic climate change, which seems to be particularly worrying as it is restricted to the uppermost elevations in its current range.

Within this study we combine molecular analyses based on cpDNA sequences and Amplified Fragment Length Polymorphisms (AFLPs) with species distribution modelling (SDM) to (1) infer the phylogeographic structure of *G. crispata* within the main distribution range on the Balkan Peninsula; (2) infer the phylogeographic relationship of the isolated Italian population to those of the Balkan Peninsula; and (3) identify potential refugial areas under past (LGM) and assumed future (global warming-induced) climatic conditions.

## Material and methods

### The study system

*Gentianella crispata* (Gentianaceae) is a monocarpic, biennial plant with an assumedly mixed mating system (Gargano et al. 2009) and low dispersal capabilities as shown for morphologically similar taxa of *Gentianella* sect. *Gentianella* (Verkaar et al. 1983). The species’ centre of distribution is located in the central to southern Dinarides, extending (i) northwards to the Plješivica mountains in southern Croatia - occurrences reported from further N given in Flora Croatia Database (Nikolić 2005 onwards) are to the best of our knowledge erroneous; (ii) southwards to Albania and North Macedonia with an isolated, southernmost population on Mt. Olympus; and (iii) eastwards to Mt. Ali Botush (Gotsev Vrah) along the border between Bulgaria and Greece. Additionally, a single population occurs in Calabria (Italy). *Gentianella crispata* depends on semi-natural to natural grassland communities from the upper montane to the alpine vegetation belt (Hartvig 1991). On the Balkan Peninsula it is mostly found in communities of the orders *Seslerietalia juncifoliae* and *Crepidetalia dinaricae* on shallow, basic soils (cf. Hruševar and Mitić 2015), but also in *Seslerietalia comosae* communities on deep, acidic soils (cf. Barudanović et al. 2007) [all in class *Elyno-Seslerietea*]. Similarly but restricted to shallow basic soils, in Italy the species usually occurs in *Seslerio nitidae-Brometum erecti* communities dominated by the two eponymous grasses (Gargano et al. 2009) but was also found in more open, stony grassland communities dominated by *Sesleria juncifolia* s.l. (Di Pietro and Wagensommer 2014). These communities are frequently shaped by traditional grazing typically with sheep (Török et al. 2018). Due to its limited geographical range *G. crispata* is listed as critically endangered in Italy (Rossi et al. 2013). On the Balkans it is considered vulnerable in Bosnia and Herzegovina (Crvena lista 2013), endangered in Kosovo (Millaku et al. 2013) and critically endangered in Bulgaria (Petrova and Vladimirov 2009). In contrast, *Gentianella crispata* remains unmentioned in the red list of rare and threatened species in Greece (Phitos et al. 1995), although it is known only from two localities along the N border and one highly isolated population on Mt. Olympus (Hartvig 1991).

### Sampling

Leaf material of 10 individuals was sampled at 15 populations of *G. crispata* covering the entire distribution area of the species plus one Balkan population of *Gentianella anisodonta* (Borbás) Á. Löve & D. Löve as outgroup for cpDNA analyses. The number of 10 samples per population is owed to the overall small size of some of the southern populations and to the attempt not to sample offspring of the same individual several times. We, however, failed to obtain *G. crispata* samples from the littoral Dinarides of Biokovo or Orjen mountains. Each population was documented with herbarium vouchers, which were deposited in the herbarium of the University of Vienna, Austria (WU). Corresponding label information including links to the Virtual Herbaria JACQ (JACQ Consortium 2004) is given in Table 1.

**Table 1.** General description of sampling localities and AFLP-derived parameters of 15 *Gentianella crispata* populations used to uncover the phylogenetic structure of the species plus one *G. anisodonta* population used as outgroup.

Sampled populations			AFLP-derived parameters								
Country (ISO code): locality (population ID)	taxon	herbarium number	individuals investigated	number of fragments	% of polymorphic fragments	Nei’s gene diversity	Shannon’s information index	PI (standard deviation)	GLD (standard deviation)	private fragments	DW
BA: Veliki Šator (1)	*G. crispata*	WU 0106530	10	149	27.0	0.082	0.125	55.333 (26.472)	0.100 (0.055)	8	75.218
BA: Troglav (2)	*G. crispata*	WU 0072925	8	122	22.1	0.075	0.113	50.821 (24.682)	0.092 (0.051)	6	82.074
BA: Vranica (3)	*G. crispata*	WU 0072850	7	182	33.0	0.108	0.164	76.667 (37.740)	0.139 (0.079)	4	81.513
BA: Čvrsnica (4)	*G. crispata*	WU 0106533	10	191	34.7	0.120	0.179	75.222 (35.473)	0.137 (0.073)	2	101.389
BA: Maglić (5)	*G. crispata*	WU 0072923	8	234	42.5	0.136	0.207	97.107 (46.849)	0.176 (0.097)	3	80.166
ME: Prutaš (6)	*G. crispata*	WU 0106537	8	250	45.4	0.146	0.222	103.357 (49.843)	0.188 (0.103)	2	74.031
ME: Savin Kuk (7)	*G. crispata*	WU 0106535	10	248	45.0	0.136	0.210	92.333 (43.465)	0.168 (0.089)	2	70.496
ME: Međukomlje (8)	*G. crispata*	WU 0106529	9	170	30.9	0.091	0.140	62.778 (29.988)	0.114 (0.062)	0	35.977
ME: Kom Vasojevićky (9)	*G. crispata*	WU 0072848	10	229	41.6	0.132	0.200	84.622 (39.863)	0.154 (0.082)	1	46.053
ME: Visitor (10)	*G. crispata*	WU 0106538	9	152	27.6	0.083	0.128	54.722 (26.183)	0.099 (0.054)	1	30.731
ME: Maja e Preht (11)	*G. crispata*	WU 0106539	10	190	34.5	0.105	0.160	69.778 (32.910)	0.127 (0.068)	4	65.712
XK: Gjeravica (12)	*G. crispata*	WU 0106528	10	104	18.9	0.057	0.088	35.422 (16.882)	0.064 (0.035)	0	37.561
BG: Tzena (13)	*G. crispata*	WU 0106522	9	100	18.1	0.063	0.095	42.111 (20.227)	0.076 (0.042)	6	80.233
BG: Gocev Vrah (14)	*G. crispata*	WU 0072900	9	108	19.6	0.066	0.099	41.889 (20.122)	0.076 (0.041)	2	42.767
IT: Dolcedorme (15)	*G. crispata*	WU 0106521	6	151	27.4	0.095	0.143	64.533 (32.615)	0.117 (0.068)	1	75.828
HR: Vaganski Vrh (016)	*G. anisodonta*	WU 0072831									

Herbarium numbers include links to Virtual Herbaria JACQ database giving detailed information on the localities, PI gives the mean number of pairwise differences between individuals and standard deviation, GLD the average gene diversity over loci and standard deviation and DW the frequency down weighted marker values.

**Figure 1.**
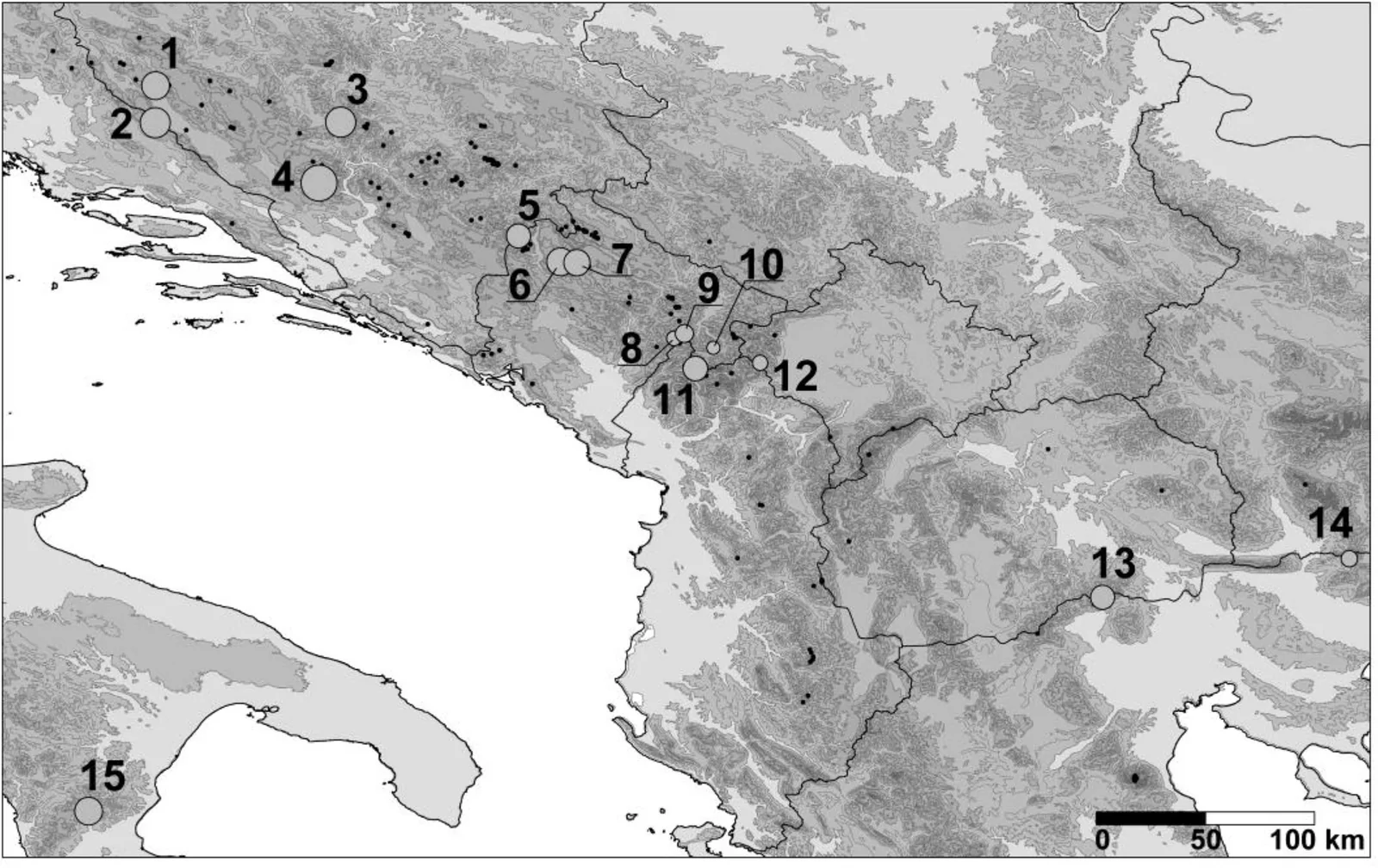
Amphi-adriatic distribution of *Gentianella crispata*. Populations used for SDMs only are indicated by black dots; those used also for molecular analyses by larger grey dots, respectively, the size of these dots is proportional to the rarity index (DW value). Numbers represent population IDs (see Table 1).

For the SDM analyses, additional occurrence records were obtained from vouchers in the herbaria LJS, MCF, NHMR, P, SO, UPA, W, WU, ZA and ZAHO, (abbreviations following Thiers 2019), as well as from published literature and hitherto unpublished field observations (see Supporting Information Table S1 and Figure 1). Combining these data with our samples, we mapped 126 occurrence sites to depict the entire distribution area of the taxon in ArcMap 10.4.1. (ESRI, Redlands, CA, USA).

### DNA extraction

Total genomic DNA was extracted from similar amounts of silica-dried leaf tissue following the CTAB protocol (Doyle and Doyle 1987) with minor modifications (Stadler et al. 2010). Quality of the extracted DNA was checked on 1% TAE agarose gels. Six blank samples were used as negative control to test for systematic contamination and 13 samples (10%) were extracted twice to be used as repeats for testing the reproducibility of AFLP fragments.

### cpDNA sequences

Two regions of the plastid genome, trnQ^UUG^- rps16 and rpl32 - trnL^UAG^ intergenic spacers were sequenced in eight individuals per population. To amplify the fragments primers of Shaw et al. (2007) were used; for a detailed laboratory protocol see Supporting Information Table S2. In few samples, sequences failed to amplify also after repeating the procedure, these were excluded ending up with six to eight individuals per population. GenBank numbers are given in Supporting Information Table S3.

### Amplified fragment length polymorphisms (AFLPs)

An initial primer trial with twelve primer combinations was conducted and three primer combinations, EcoRII (6-FAM) ACA - MseI CAT, EcoRII (VIC) AAG - MseI CTG and EcoRII (NED) AAC - MseI CAT, were chosen. The procedure followed Vos et al. (1995) with modifications as in Schönswetter et al. (2009). Purification followed Schönswetter et al. (2009) with the modification that 2 μL of each selective PCR product were directly eluted into 10 μL formamide (Applied Biosystems) and 0.1 μL GeneScan ROX (Applied Biosystems, Foster City, CA, USA).

Eleven samples where processed twice to allow an assessment of the reproducibility of results by calculating an error rate. AFLP fragment scoring was performed with GeneMapper software version 3.7 (Applied Biosystems). Fragments were automatically binned and manually revised. Bins were accepted if bands fulfilled the following criteria: (i) band intensity clearly above the background noise; (ii) smoothly curved peaks; and (iii) bin width not above 0.8 base pairs (bp) to secure homology of bands in a bin. Fragments were scored between 50 and 500 bp. Following Bonin et al. (2004), for each pair of replicated samples a ratio of the number of fragments matching and mismatching was calculated. Fragments with a reproducibility below 90% and monomorphic fragments were excluded from further analyses. In few samples, the fragments failed to amplify also after repeating the procedure, these were excluded ending up with six to ten individuals per population.

### Analyses of cpDNA data

All sequences were assembled with SEQMAN II (DNAStar, Madison, WI, USA), edited manually and aligned using BIOEDIT 7.1.3.0 (Hall 1999). Assuming the chloroplast genome is a single linkage group, the sequences were concatenated and edited: Indels longer than 1 bp were coded as single events and length variation in mononucleotide repeats were excluded (Ingvarsson et al. 2003). In TCS 1.21 (Clement et al. 2000) a haplotype network with gaps as fifth character state and a 95% connection limit was created.

Phylogenetic analyses with each haplotype represented by a single sequence and corresponding sequences of *G. anisodonta* as outgroup were performed to identify cpDNA haplotype lineages. Sequence evolution models were evaluated using the AIC by applying Modeltest 3.7 (Posada and Crandall 1998). The K81 model of nucleotide substitution (Kimura 1981) was found to provide the best fit for the sequence data. As this is not implemented in MrBayes 3.2.7 (Ronquist et al. 2012) we adopted the GTR model (Tavaré 1986), which best approximates the K81 model. Two independent analyses with 5 × 10^6^ generations and sampling every 100 generations were run, the first 30% of trees were discarded as burn-in and the remaining ones (70,002 trees) were used to build a majority rule consensus tree.

Finally, we performed a spatial analysis of molecular variance (SAMOVA) using SAMOVA 2.0 (Dupanloup et al. 2002) to infer the number of groups. This approach iteratively seeks to define groups which have a maximum genetic differentiation and are geographically homogeneous (Dupanloup et al. 2002). The number of initial conditions was set to 500 with *K* = 2-14. The most likely number of groups was inferred from the corresponding FCT, using the configuration with the highest FCT without single population groups, as group structures were not represented by configurations including single population clusters (Heuertz et al. 2004; Lee and Maki 2013).

### Analyses of AFLP data

To assess the genetic variation the following parameters were calculated for each population: AFLP-SURV 1.0 (Vekemans 2002) was used to calculate the number of fragments per population (Nfrag) and percentage of polymorphic fragments (Pfrag), PopGene 1.32 (Yeh et al. 1999) to calculate Nei’s (1987) gene diversity (H) and Shannon’s information index (S) with the assumption of Hardy-Weinberg equilibrium. Arlequin ver 3.5 (Excoffier et al. 2005) was employed to calculate the mean number of pairwise differences between individuals (PI) with the number of permutations set to 1000 and the average gene diversity over loci (GDL). GeneAlEx 6.5 (Peakall and Smouse 2006) was used to calculate the number of private fragments (Priv). Frequency down weighted marker values per population (DW) (Schönswetter and Tribsch 2005) were calculated as rarity index to depict population genetic divergence using the package AFLPdat (Ehrich 2006) in R 3.6.3 (R Core Team 2020) with six randomly chosen individuals per population to avoid effects of uneven sample sizes. The DW values are expected to be high in isolated populations and low in recently colonized areas, suggesting either vicariance or recent migration (Schönswetter and Tribsch 2005).

Nei-Li distances (Nei and Li 1979) derived from TreeCon 1.3 b (Van de Peer and De Wachter 1994) were used to build a neighbor-net diagram by applying SplitsTree 4.14.6 (Huson and Bryant 2006). In addition, a neighbor-joining analysis bootstrapped with 1000 pseudoreplicates was performed using TreeCon to evaluate support for the major branches of the neighbor-net.

A principal coordinate analysis (PCoA) based on a Euclidean genetic distance matrix was performed using adegenet v2.1.1 (Jombart and Ahmed 2011).

A Bayesian model-based cluster analysis was performed with STRUCTURE 2.3.4 (Pritchard et al. 2000; Falush et al. 2007) to identify genetic groups. K was set from 2 to 15 with 10 replicates for each K, an admixture model and independent allele frequencies among populations were assumed. In each run a burn-in period of 5 × 10^4^ iterations was followed by 5 × 10^5^ MCMC iterations. Results were analysed in the online tool Structure Harvester (Earl and Holdt 2012) and the most likely K was evaluated following Evanno et al. (2005). Finally, the best alignment of the clusters was calculated employing CLUMPP 1.1.2 (Jakobsson and Rosenberg 2007) and the final data was visualized using Distruct 1.1 (Rosenberg 2003).

Additionally, Arlequin ver 3.5.1.3 (Excoffier et al. 2005) was used to perform non-hierarchical (i.e. without grouping populations) and hierarchical analyses of molecular variance (AMOVA) with the number of permutations set to 1000. For the hierarchical approach various groupings based on the STRUCTURE analysis of the AFLP data and the SAMOVA analysis of the cpDNA data were used. For details regarding the allocation of populations to groups see Table 2.

**Table 2.** AMOVA based on AFLP data of *Gentianella crispata* samples (1) applying no additional grouping but populations; (2) groups defined according to STRUCTURE results with admixed populations assigned to the southern group (populations 8-15); (3) admixed populations assigned to northern group (populations 1-4); (4) admixed populations kept as separate group; (5) groups defined according to SAMOVA results with three clusters.

	**Grouping**	N	Source of variation	*df*	SS	Variance [%]	Fixation index
**(1)**	according to populations	15	Among populations	14	5023.5	52.6	*F*_st_ = 0.526
			Within populations	117	3906.4	47.4	
**(2)**	[1, 2, 3, 4]	2	Among groups	1	1225.1	22.8	
	[5, 6, 7, 8, 9, 10, 11, 12, 13, 14, 15]		Among populations within groups	13	3798.4	36.2	*F*_st_ = 0.589
			Within populations	117	3906.4	41.0	
**(3)**	[1, 2, 3, 4, 5, 6, 7]	2	Among groups	1	1134.2	16.6	
	[8, 9, 10, 11, 12, 13, 14, 15]		Among populations within groups	13	3889.4	39.7	*F*_st_ = 0.880
			Within populations	117	3906.4	43.7	
**(4)**	[1, 2, 3, 4]	3	Among groups	2	1814.1	21.3	
	[5, 6, 7]		Among populations within groups	12	3209.4	34.9	*F*_st_ = 0.562
	[8, 9, 10, 11, 12, 13, 14, 15]		Within populations	117	3906.4	43.8	
**(5)**	[1, 2, 3, 4]	3	Among groups	2	1790.1	21.8	
	[5, 6, 7, 9, 8, 10, 11, 13, 15]		Among populations within groups	12	3233.4	35.0	*F*_st_ = 0.568
	[12, 14]		Within populations	117	3906.4	43.2	

Populations of the same group are enclosed by a bracket. N gives the number of groups, df the degrees freedom, SS the mean sum of squares, Fst the fixation index. p values of all analyses were < .001.

### Bioclimatic variables

Temperature and precipitation data from the Chelsa Climate database (Karger et al. 2017a, 2017b), available with a spatial resolution of 30“ at http://chelsa-climate.org/, was used to identify suitable areas under historical, current and future climatic conditions. To represent future climatic conditions an IPCC5 scenario from the Representative Concentration Pathways-family representing intermediate climate change (RCP45) was chosen. To consider model uncertainties we used climate predictions derived from three Global circulation models (MRI-CGCM3, FGOALS-g2, CNRM-CM5) covering both, historical (i.e. LGM, 21000 BP) as well as future (i.e. 2061-2080) climatic conditions.

We used three bioclimatic variables (|Pearsons *r*| < 0.6) relevant for high elevation plant species (Dullinger et al. 2012): Temperature Annual Range (bio7), Mean Temperature of Warmest Quarter (bio10) and Precipitation of Driest Quarter (bio17). These climatic variables were projected to a grid of 1 × 1 km cell size using the nearest neighbour method.

### Modelling occurrence probability

We used species distribution models (SDMs) to project suitable areas of *G. crispata* under past, current and future climatic conditions. These models relate species’ occurrences (presences/absences) to the three environmental variables described above at a resolution of 1 km. To do so, 126 sampled populations (see ‘Sampling’) were used as presences, while 126 pseudo-absences were randomly drawn from a 250 km buffered rectangle around the species presences. Following adapted recommendations by Barbet-Massin et al. (2012) selection of pseudo-absence was repeated ten times. SDMs were parameterized within the BIOMOD2 framework (Thuiller et al. 2016) by means of two modelling techniques using their default settings: Boosted Regression Trees (GBM) and Random Forests (RF). Model runs were replicated three times (for each set of pseudo-absences) using 80% of data for model parameterization and the remaining 20% for model evaluation using the True Skill Statistic score (TSS) (Allouche et al. 2006). Based on the resulting 60 parameterized models (3 × 10 for each modelling technique), we calculated ensemble projections of potential species ranges under current, historical (i.e. 17,100 BP) and future (i.e. 2080) climatic conditions as weighted (by the TSS value of single models) mean of the projected occurrence probabilities of the single models. Finally, probability values of these projections based on the three GCMs were averaged to achieve a single continuous consensus prediction for historic as well as future conditions. These consensus projections were further translated into binary predictions (suitable/unsuitable) using the threshold that maximises the TSS value (Liu et al. 2005).

## Results

### Genetic evidence - cpDNA

**Figure 2.**
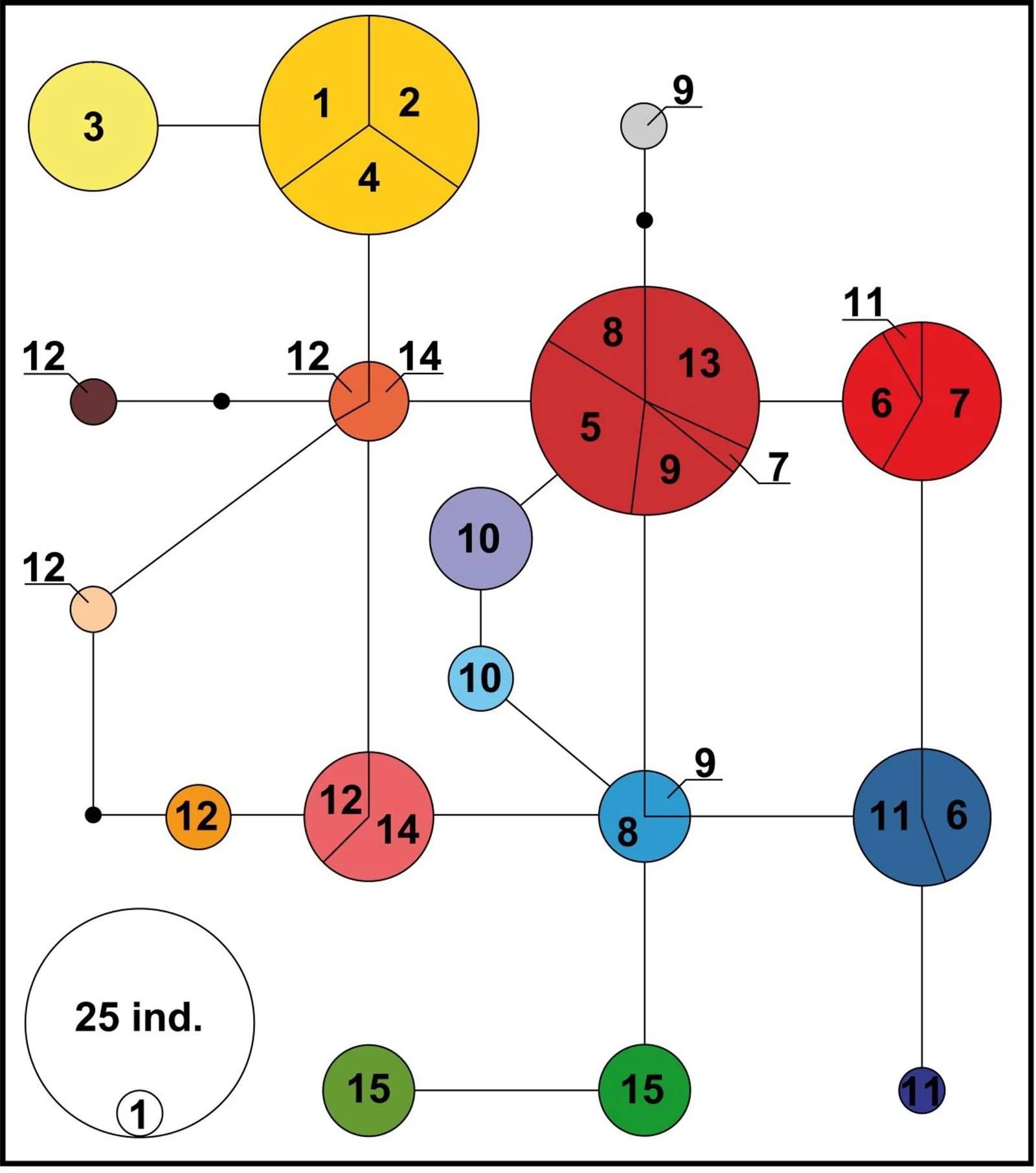
Haplotype network derived from the concatenated plastid sequences of trnQ^UUG^- rps16 and rpl32 - trnL^UAG^ intergenic spacers of *Gentianella crispata* samples, numbers represent population IDs (see Table 1). The area of circles representing haplotypes is proportional to the number of individuals sampled belonging to that haplotype (range between one and 25 individuals as shown by the white circles in the lower left corner).

**Figure 3.**
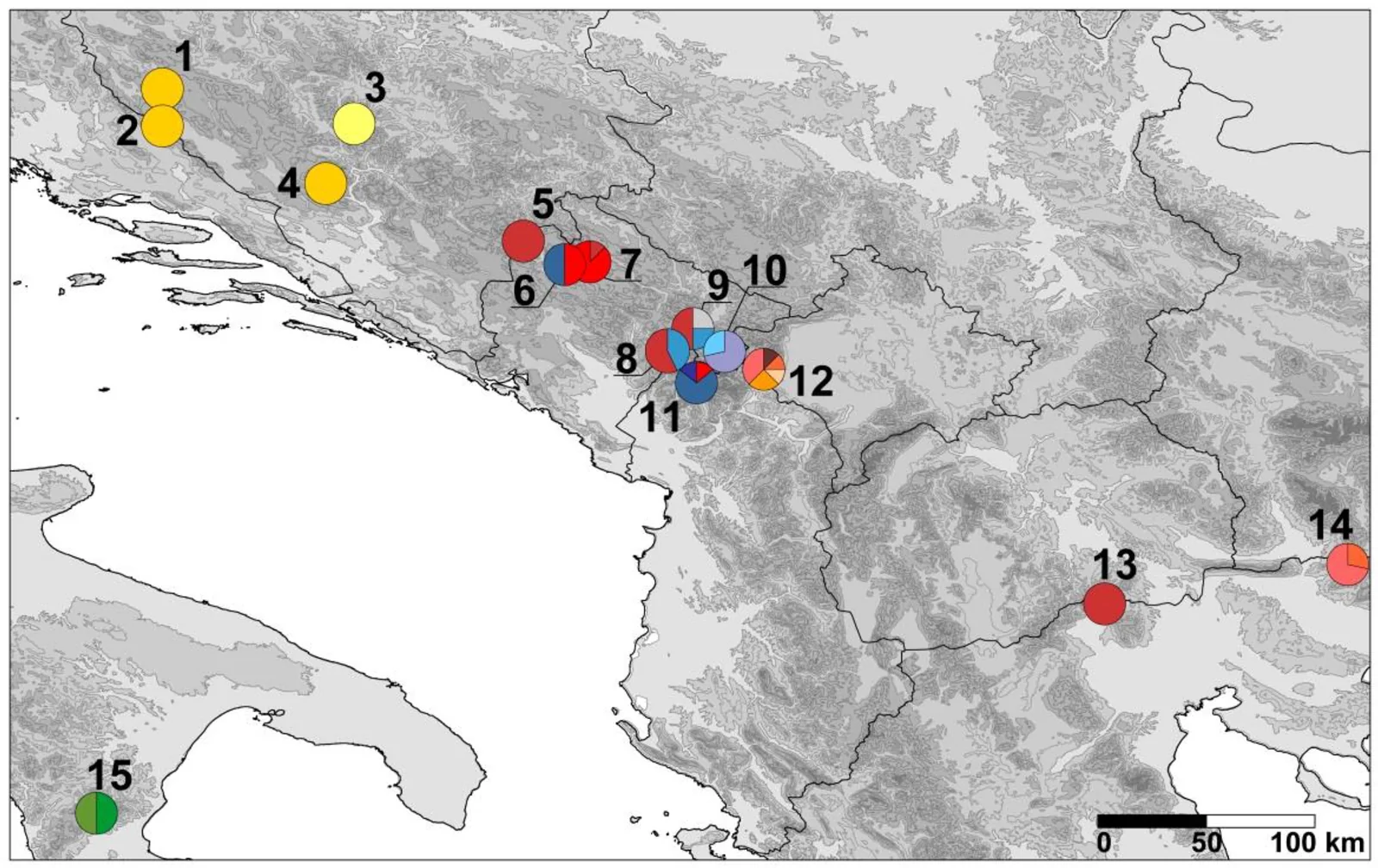
Geographic distribution of haplotypes derived from the concatenated plastid sequences of trnQ^UUG^- rps16 and rpl32 - trnL^UAG^ intergenic spacers of *Gentianella crispata* samples, colour-coding according to the haplotypes as in Figure 2, numbers represent population IDs (see Table 1). Segments of pie charts in populations exhibiting two or more haplotypes are proportional to the number of individuals carrying the haplotype.

The rps16-trnQ and the rpl32-trnL intergenic spacer have a length of 721 bp and 610 bp, respectively. The alignment is 1336 bp long and comprises 13 variable sites. In general, the haplotype network (Figure 2) shows only shallow spatial resolution (Figure 3). 17 haplotypes were identified in 113 individuals, variation within populations was found in populations 6, 7, 8, 10, 14, 15 (two haplotypes), populations 9, 11 (three haplotypes) and in population 12 (five haplotypes). Ten haplotypes are restricted to one population each, seven are found in two or more, the most frequent one occurs in five populations. The northernmost populations (1, 2, 3, 4) are separated with populations 1, 2 and 4 sharing one large haplotype and population 3 having a unique haplotype connected to the first one. The central and the southern Dinaric populations shared haplotypes, too; one haplotype was found in populations 7, 8 and 9 from the Dinarides as well as all individuals of populations 5 (again from the Dinarides) and 13 (from the North Macedonian/Greek border). The easternmost population 14 shares two haplotypes with population 12 from the southern Dinarides. The single Italian population, 15, exhibits two unique haplotypes which are connected to a haplotype shared by individuals of populations 8 and 9.

In the Bayesian majority rule consensus tree (Supporting Information Figure S2) population 15 appears in a large polytomy containing populations 9 and 10 as well as other populations of the southern Dinarides.

The SAMOVA simulations suggested a clear increase of the FCT values from *K* = 2 to *K* = 8 where it reached a plateau. The highest FCT value was reached with *K* = 12, however, with a high number of single population clusters (Supporting Information Table S4). The configuration comprising the highest FCT but lacking single population clusters is represented by three clusters constituted by (i) the northernmost populations (1, 2, 3, 4), (ii) the central and southern populations including the Italian one (5, 6, 7, 8, 9, 10, 11, 13, 15) and (iii) one southern and one south-eastern population (12, 14). 55.82% of haplotype variation was found among the clusters, 30.89% among populations within clusters and only 13.29% within populations.

### Genetic evidence - AFLPs

We scored 631 AFLP fragments ranging from 57 to 497 base pairs in 133 individuals (6-10 per population), 11 fragments (1.7%) were monomorphic, the error rate was 2.1%. All AFLP diversity indices are given in Table 1, the geographic distribution of populations with respect to DW values is depicted in Figure 1, low values are aggregated in the southern Dinarides.

**Figure 4.**
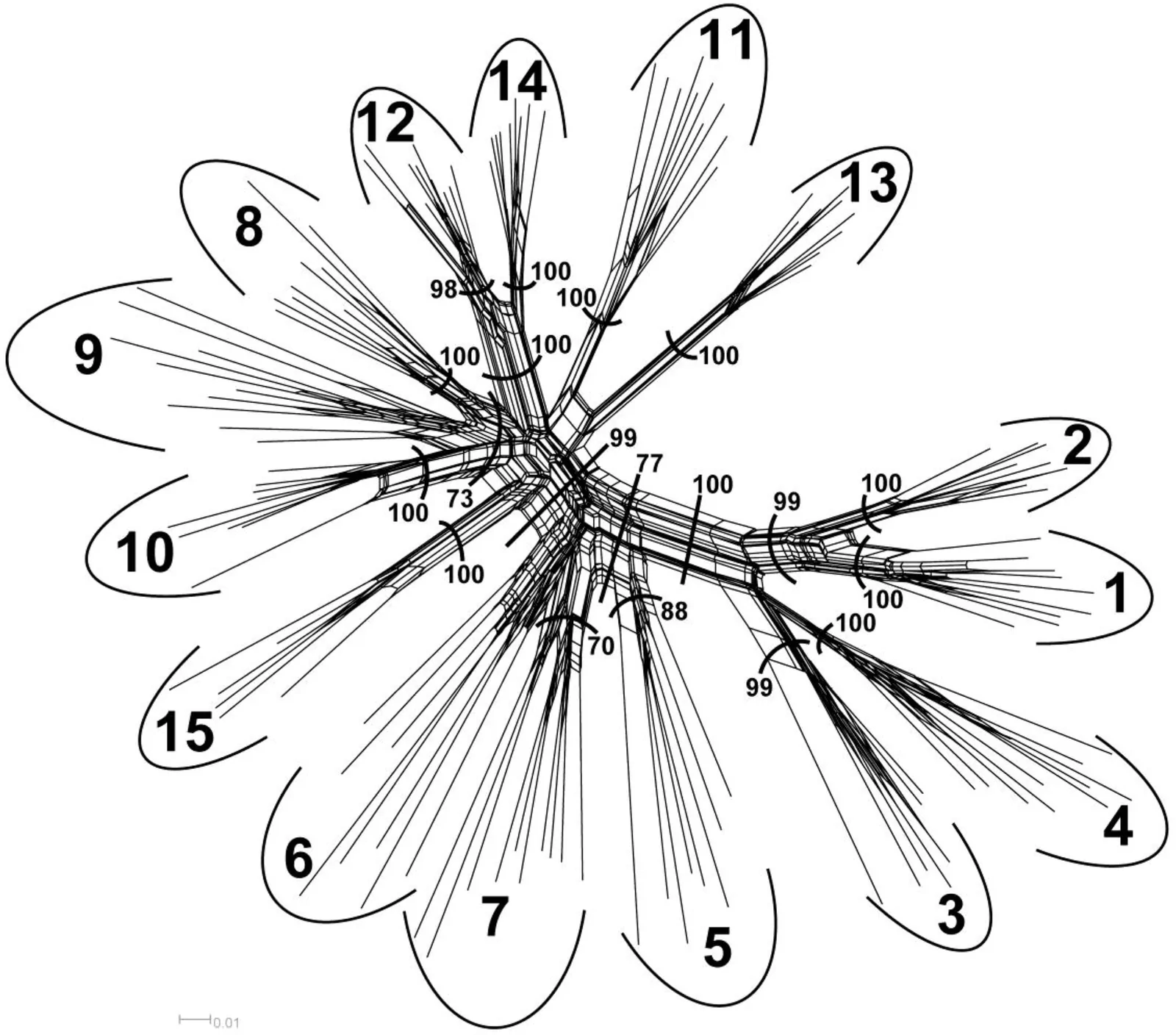
Neighbor-net with bootstrap values for major branches (≥70% shown) obtained from AFLP data of *Gentianella crispata* samples. Numbers represent population IDs (see Table 1).

The neighbor-net (Figure 4) shows a clear split between the northernmost populations (1, 2, 3, 4) and the remaining ones. Within the latter we found a well-supported separation between the central Dinaric populations in northern Montenegro (5, 6, 7) and the southern Dinaric populations (8, 9, 10, 11, 12) including the isolated south-eastern populations (13, 14) and the Italian population (15). All populations except population 6 (associated with spatially close population 7) and population 9 (associated with populations 8 and 10) form well-supported groups (bootstrap > 70) on their own.

The PCoA (Supporting Information Figure S1) largely confirms the results of the neighbor-net by showing a clear split between the northernmost populations (1, 2, 3, 4) and the remaining ones. The Italian population (15) appears connected to the southern Dinaric populations (except population 12) including population 13, from which a cluster with the central Dinaric populations (5, 6, 7) and another one with populations 12 and 14 is separated.

**Figure 5.**
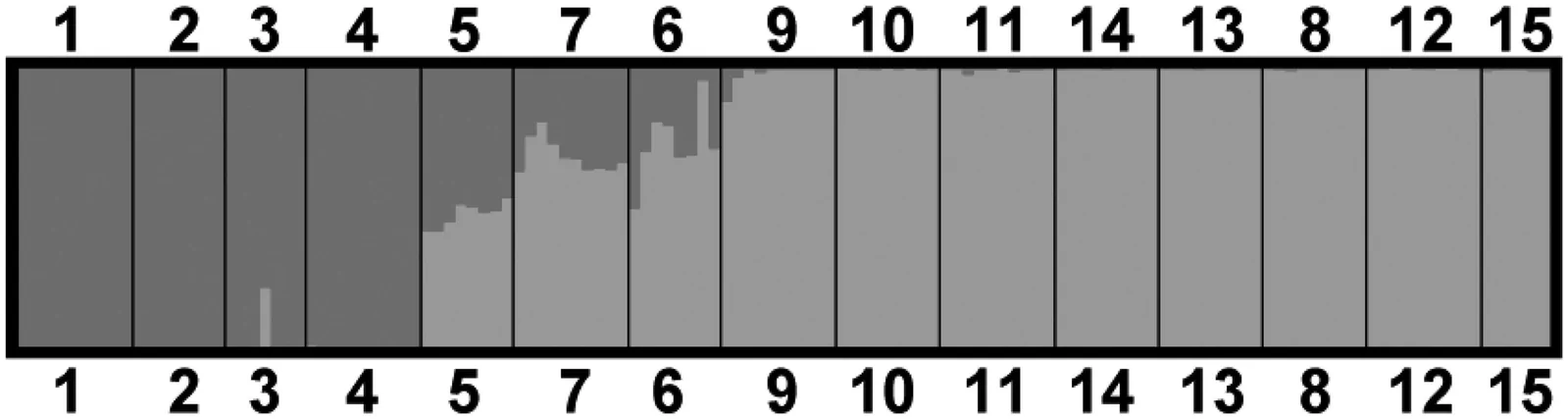
Bar graph showing the genetic structure of the investigated *Gentianella crispata* populations based on AFLP data and assessed with the software STRUCTURE, each of the two groups is represent by a colour.

The STRUCTURE analysis proposed *K* = 2 as the appropriate number of groups. The northernmost populations (1, 2, 3, 4) form the first group, while the remaining populations belong to the second group. The central Dinaric populations (5, 6, 7), however, show admixture between these groups (see Figure 5).

Results of AMOVA analyses were highly congruent, finding 40 − 50% of the overall genetic variation to be within populations. In the hierarchical AMOVAs variation among groups was around 20% for all groupings of populations (Table 2).

### Species distribution models

**Figure 6.**
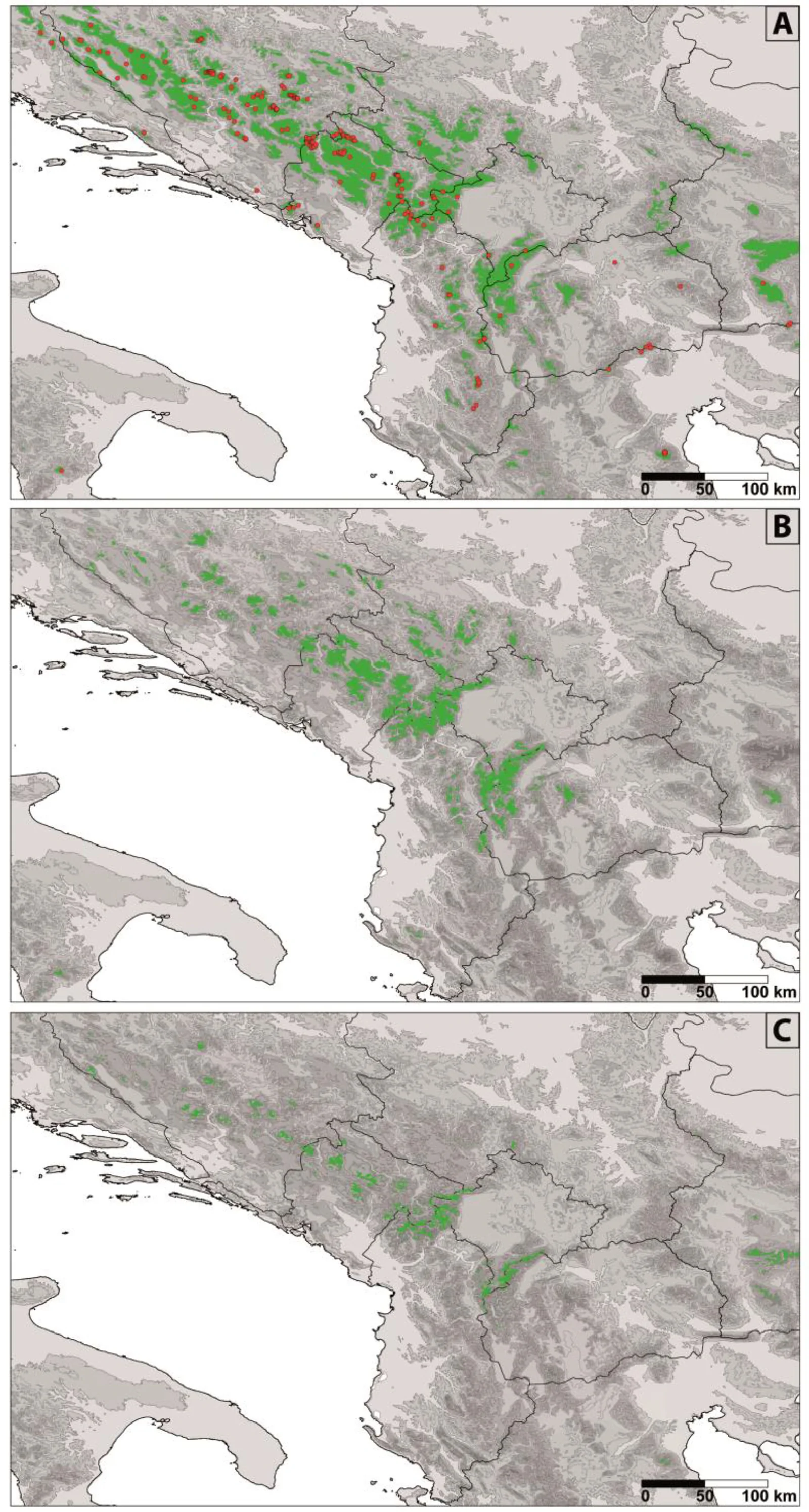
Species distribution models based on current *Gentianella crispata* distribution data, binary map with suitable areas highlighted in green: (A) recent distribution of suitable areas including known current occurrences of *G. crispata* symbolized by red dots, (B) hindcast of suitable areas during LGM, (C) forecast of suitable habitats under moderate future climate warming conditions at 2080.

SDM predictions of suitable areas under current climatic conditions match the actual distribution very well (Figure 6A). Nearly all recent presences are located in a suitable area including the southernmost populations in northern Greece and the isolated population in Italy (except for some populations at relatively low elevations: in Bosnia-Herzegovina close to the Croatian border and eastern North Macedonia). SDMs predict a severe reduction of suitable area under both, past and future climatic conditions. At the LGM the predicted distribution of *G. crispata* is more fragmented along the main range of the Dinarides. More peripheral parts of the current range were entirely unsuitable; in the littoral Dinarides only the highest part of the Orjen mountains was found the offer suitable habitats (Figure 6B). A similar pattern can be observed at 2080: the distribution of *G. crispata* is predicted to be even more fragmented in the central Dinarides, while the southernmost populations and the populations in the littoral Dinarides are predicted to be lost except for the Mt. Olympus one (Figure 6C). At the LGM and 2080 the larger area around the current population in Italy is predicted to be suitable and unsuitable, respectively. Continuous distribution models are shown in Supporting Information Figure S3.

## Discussion

### The Balkan core area

On the Balkan Peninsula we found a northern cluster of *Gentianella crispata* populations (1-4) clearly separated from those of the central and southern Dinarides. The combined evidence (high DW values, distinct haplotypes, low haplotype diversity, well-supported group in the Bayesian majority rule consensus tree) indicates that these northernmost populations were isolated from the central and southern Dinaric populations already prior to or during the Pleistocene in a northern refugium. This supports the refugia-within-refugia concept (Gómez and Lunt 2007) as found in other plant species of the Balkan Peninsula (e.g. Surina et al. 2011; Jug-Dujaković et al. 2020). The observed strong phylogeographic split follows the Neretva river, which provides an effective barrier along its narrow canyon as found in a number of other case studies on both animals (e.g. lizards, Podnar et al. 2014; voles, Kryštufek et al. 2007) and high mountain plants such as *Cerastium dinaricum* Beck & Szyszył. (Kutnjak et al. 2014), lowland plants, e.g. *Edraianthus tenuifolius* (Waldst. & Kit.) A. DC. (Surina et al. 2011) and the *Campanula pyramidalis* group (Lakušić et al. 2013) as well as plants occurring along a broad altitudinal gradient, e.g. *Euphorbia myrsinites* L. (Falch et al. 2019). Several reasons have been proposed for this barrier. Lakušić et al. (2013) emphasized climatic reasons due to increased continentality in areas close to the dry northern Adriatic basin during cold stages of the Pleistocene, whereas Falch et al. (2019) proposed strong genetic drift in populations re-immigrating northwards. In contrast, this genetic break along the Neretva river could not be observed in the cold-adapted high-mountain plant *Edraianthus serpyllifolius* (Vis.) A. DC. (Surina et al. 2011). In *G. crispata*, a mountain taxon with low dispersal capabilities, we assume the topography to function as a barrier leading to the separation of a northern refugium. In the orographically complex landscapes of the species’ range, mountain systems are typically separated by deep valleys with thermophilic vegetation restricting migration and gene flow between populations. This is also reflected by most populations of our sample forming distinct genetic entities as seen in the AFLP data (Figure 4).

Accordingly, the predicted pattern of climatically suitable areas during LGM can be interpreted in terms of the refugia-within-refugia concept. SDMs hindcast larger areas of potential refugia along the northern margin of the species’ current distribution, whereas refugia become more constricted and more scattered southwards, clearly leading to an isolation of the central Dinaric populations (Figure 6B). The fact, that the hindcast based on present-day ecological niches did not identify suitable areas within the southern part of the species’ Balkan range can probably be attributed to drier climate conditions during the LGM (Willis 1994), but does not exclude possible southern refugia. A comparable distribution pattern of suitable areas during LGM was found for the Mediterranean lowland plant *Edraianthus tenuifolius*. In this case SDMs predicted suitable regions even further in the north, however supplemented by a disjunct refugium in the south, mainly in Montenegro and northern Albania (Glasnović et al. 2018). In *Salvia officinalis* L., another lowland plant, one refugium in the range of the southernmost Dinaric populations was found. In this case, depending on the type of predictive model, two additional refugia further north were, or were not, identified (Rešetnik et al. 2016a).

The central Dinaric populations are located at Mt. Maglić and the Durmitor mountain range in north-western Montenegro. This biogeographically intermediate position indicates the area as a secondary contact zone between the four separated northern populations and the southern Dinaric populations (8-12) emerged due to post-glacial range expansions (Dapporto 2010; Leroy et al. 2017). There are, however, no shared plastid haplotypes between these two groups. cpDNA is maternally inherited in Gentianaceae (Harris and Ingram 1991) and pollinator-mediated southwards pollen transfer over such large distances is considered implausible in *Gentianella* (Paland and Schmid 2003). Other features like the increased genetic diversity within the admixed populations could be a result of a secondary contact between lineages previously isolated due to their persistence in distinct refugia. Increased DW values, conversely, would suggest a period of isolation also from the southern cluster, in spite of the occurrence of shared haplotypes.

Relationships among the southern Dinaric populations are poorly resolved (Figures 2 and 4 and Supporting Information Figure S1). While for populations 8-10 from the Komovi mountain range and Mt. Visitor in south-eastern Montenegro we can assume post-glacial recolonization of this part of the Dinarides, the isolated position and high rarity value of population 11 hint at a possible refugial area in the Prokletije, an isolated mountain system with a high number of relict and endemic taxa (Rakaj 2009).

Population 13 located in the Tzena massif (border between North Macedonia and Greece) and population 14 at the Slavyanka mountains (border between Bulgaria and Greece) represent isolated outposts at the south-easternmost edge of the known distribution. Both areas are considered hotspots of biodiversity and centres of endemism and relict species (Stevanović et al. 2007). The high rarity value of population 13 (Table 1) hints at a long time *in situ* survival although this population shares a monomorphic haplotype with central and southern Dinaric populations (Figures 2 and 3). In contrast, population 14 exhibiting a low rarity value appears surprisingly linked to population 12 from Prokletije mountains in the southernmost Dinarides (Figures 2 and 4 and Supporting Information Figure S1) as evidenced by both genetic markers. The few extant occurrences in North Macedonia and Bulgaria connecting population 14 and 12 probably represent the thinning remnants of a more continuous range at the rear edge of the species´ distribution. Given the lack of specific adaptations renders the likelihood for LDD very low. Low levels of genetic diversity in populations 12-14 indicate restricted gene flow between marginal populations, again a pattern typical for the rear edge at the southern margin of the distribution area (Hewitt 2000; Petrova et al. 2018). Rear edge populations may potentially be well adapted to stressful environments and play an important role in conserving the genetic variation of a species (Hampe and Petit 2005), notably also in the context of adaptation to human-mediated climate change (Razgour et al. 2013).

Based on material from the Komovi mountains Bjelčić and Mayer (1973) separated a small-flowered taxon, *Gentianella pevalekii* Bjelčić & E. Mayer, from *G. crispata*. Both taxa, however, occur in sympatry according to Bjelčić and Mayer (1973) and Stevanović and Jakovljević (2014). Sampling from the highest elevations of the respective mountain system we could not find plants with distinctly smaller flowers and our genetic data do not support a taxonomic differentiation. Furthermore, Bjelčić and Mayer (1973) discriminated three pseudo-seasonal ecotypes as variants within *G. crispata*, however, they also noted transitions between the three morphs.

### The Italian connection

Both genetic markers (cpDNA and AFLPs) show a close connection of the only known Italian population of *Gentianella crispata* to the central Dinaric and southern Dinaric populations (Figures 2 and 4, Table 2) of southernmost Bosnia and Herzegovina, Montenegro and north-western Kosovo. A similar source area, i.e. Montenegro and northern Albania, was suggested for the Italian populations of *Euphorbia myrsinites* (Falch et al. 2019). Among other taxa showing a similar amphi-adriatic pattern *Genista sericea* Wulfen var. *rigida* Pamp. shows a strong genetic coherence between S Italy and the Dinarides and a clear separation from northern peri-adriatic samples of its close relative *G. sericea* s.str. (Vižintin et al. 2012). Similarly, in *Edraianthus graminifolius* the most pronounced genetic split was not found between the Balkan and the Apennine Peninsula but within the western Balkan (Surina et al. 2014). This Balkan split most likely results from Pleistocene isolation (see above). Thus, early migration events such as during the late Pliocene are very unlikely in the case of *G. crispata* for two reasons:

i *G. crispata* is a relatively young taxon considering the age estimates in Bernhard von Hagen and Kadereit (2001) with the split between the European fimbriate and efimbriate lineages (e.g. *G. aurea*, N-Eur) occurring between 2.6 my ± 0.8 my and 4.9 my ± 1.4 my ago. So this fundamental split into fimbriate and efimbriate *Gentianella* occurred most likely in the Pliocene. The mean ML pairwise genetic distance estimate for a basal split among the Eurasian fimbriate species based on ITS and mat*K* data, however, did not allow a reasonable age estimate. From these data, one can only conclude that lineage splitting in European fimbriate *Gentianella* started some time at the end of the Pliocene or early Pleistocene.
ii The main genetic split detected by both genetic markers was found on the Balkan Peninsula, not between the Balkans and Italy. Migration to the Apennine Peninsula during or after the Pleistocene appears most likely. Trans-adriatic dispersal over land bridges in the northern Adriatic Sea during cold periods of the Pleistocene (Correggiari et al. 1996) could have occurred. However, in this case we would expect a more marked genetic differentiation, the existence of more than one Italian population and a Balkan source area further north. Such patterns were observed, e.g. in the *Euphorbia barrelieri* group (Frajman and Schönswetter 2017) with the split between the Italian and Balkan representatives roughly dated at 0.6-0.2 my.

The low genetic differentiation of the Italian population, its average genetic diversity (see also Gargano et al. 2009) and increased rarity value (Table 1) would be compatible with a more recent immigration. However, the probability for dispersal by natural means is low due to lacking morphological adaptations of the seeds, but dispersal of grassland species such as *G. crispata* is likely mediated via transhumance or human migration events (Poschlod and WallisDeVries 2002; Kaligarič et al. 2016). Likewise, the genetic structure of *G. praecox* (A. Kern. & Jos. Kern.) Dostál ex E. Mayer, a close relative of *G. crispata* distributed in central Europe, could partly be explained by historical trade routes for salt and cattle (Königer et al. 2012).

Human-mediated seed dispersal seems even more likely as the Italian population of *G. crispata* is found in the Pollino National Park along the regional borders of Basilicata and Calabria in S Italy. This area is characterised by a high number of Albanian speaking linguistic enclaves (Pellegrini 1977, and map therein). Albanian settlers, who have crossed the Adriatic Sea in the fifteenth and sixteenth century, typically were shepherds and farmers (Bracco et al. 2015), who brought their domestic stock - likely together with seeds of grassland species - from the Balkan areas of origin. Thus, human-mediated plant dispersal may have contributed to the high number of amphi-adriatic plant taxa in the regions of Basilicata and Calabria (Pezzetta 2010).

Assuming such a human-mediated dispersal, the separation of the Italian population of *G. crispata* from the Balkan populations would have lasted for about 500 years representing 250 generations of this biennial species. This time period seems plausible to reach the degree of genetic divergence that has been observed in the Italian population. However, from our data we cannot exclude LDD at some earlier time.

### Future prospects

*Gentianella crispata* is a typical representative of FFH habitat type *6170 Alpine and subalpine calcareous grasslands* listed in Annex I of the European Union’s habitat directive (Council of the European Commission 1992) on the western Balkan Peninsula (Hruševar and Mitić 2015). These natural to semi-natural habitats are considered threatened throughout SE Europe (Török et al. 2018). Plant taxa constituting these mountain grasslands on the Mediterranean Peninsulas are especially endangered due to combined deteriorating effects of increased temperatures, lower precipitation and strong anthropogenic disturbance in some of the mountain ranges (Gentili et al. 2015). We predicted a substantial contraction of areas with suitable habitats for *Gentianella crispata* at 2080 compared to the present-day situation. The areas currently occupied by the species in the main range along the higher mountains of the Dinarides retain suitable habitats, but their area becomes much smaller and more fragmented (Figure 6C). This indicates that *G. crispata* will be even more restricted to the highest elevations than under current conditions. Thus, further upslope migration seems highly unfeasible especially at the southern edge of its range and it remains doubtful if adaption can keep pace with rapid climate change. In addition, the entire areas close to the Adriatic coast-line, some of the northernmost areas as well as most of the southern and south-eastern ones occupied today are predicted to become unsuitable (Figure 6C). However, in the SDM some of the actual and recently documented occurrences in North Macedonia are classified as unsuitable habitats already in current data. This may indicate local adaptation to warmer and more arid climate conditions and thus underlines the importance of the southern edge for future survival (Hampe and Petit 2005) of this and other mountain taxa of the Balkan Peninsula. Additionally, dry calcareous grasslands of FFH *6170* appear to be comparably resilient towards increase of temperature and periods of drought (Petriccione and Bricca 2019). However, these findings may be attributed to a lag behind actual climate change and extinction events are yet to be expected (Rumpf et al. 2019).

## Supplementary Information

Supplementary file (ZIP 7.61 mb)
