# Supplementary material for: Biogeography of amphi-adriatic Gentianella crispata (Gentianaceae): a northern refugium and recent trans-adriatic migration
Source: Plant Biosyst. 2021 Jun 1;156(3):754–68. doi: 10.1080/11263504.2021.1918783 (PMC13627213; doi:10.1080/11263504.2021.1918783)
Supplement: Supplementary file 1 [file 44473_2022_15603014_MOESM1_ESM.zip › Table_S1.docx]

**Table S1.** Records of *Gentianella crispata* used only for SDM analysis (i.e., not given in Table 1 and Table S2) giving the iso-coded country, locality, coordinates including uncertainty in meter and data source: either herbarium acronym following Thiers (2019), publication or pers. comm.

| **Taxon** | **Country** | **Locality_short** | **N** | **E** | **uncertainty** |
| --- | --- | --- | --- | --- | --- |
| *Gentianella crispata* | MNE | Zabljak-czernojezero 1500m | 43.147616 | 19.097313 | 500 |
| *Gentianella crispata* | MNE | Höhenrücken westlich Cakor-Paß | 42.668355 | 20.006267 | 500 |
| *Gentianella crispata* | MNE | Silikatgebiet nördlich Cakor-Paß | 42.688627 | 19.999661 | 500 |
| *Gentianella crispata* | MNE | in declivibus orientalibus sub jugo Cakor prope Plav 1700m | 42.669462 | 20.01641 | 500 |
| *Gentianella crispata* | MNE | Crna Gora. 40 Km NNE of Niksic, 7 Km SSE of Zabljak, Riblje Jezero 1400m | 43.090656 | 19.150837 | 500 |
| *Gentianella crispata* | BIH | am lisin bei ivan | 43.876778 | 17.989173 | 500 |
| *Gentianella crispata* | MNE | Bobotov Kuk - Prevoj Sedlo | 43.127739 | 19.037151 | 250 |
| *Gentianella crispata* | BGR | Gemeinde Razlog, 0,7 km südöstlich Gipfel Gushterova Primka | 41.807667 | 23.363567 | 500 |
| *Gentianella crispata* | MNE | between Kolasin and Pec, near tresnjevik pass | 42.693947 | 19.676614 | 250 |
| *Gentianella crispata* | GRC | Olymp (Olimbos) - Skala | 40.082326 | 22.357156 | 500 |
| *Gentianella crispata* | HRV | Plješivica - Bukovi Vrh | 44.354067 | 15.999618 | 500 |
| *Gentianella crispata* | HRV | Plješivica - Postak | 44.255132 | 16.110269 | 500 |
| *Gentianella crispata* | XKX | Rugova planina - Košutane (Koshutan) | 42.73745 | 20.100801 | 500 |
| *Gentianella crispata* | BIH | prokosko jezero | 43.957622 | 17.754826 | 500 |
| *Gentianella crispata* | BIH | Vranica planina | 43.951234 | 17.685792 | 500 |
| *Gentianella crispata* | BIH | Dovlici, Pukov? cajir | 43.815663 | 18.45862 | 1000 |
| *Gentianella crispata* | BIH | Bitovnja planina | 43.800815 | 17.942336 | 1000 |
| *Gentianella crispata* | MNE | Orjen - Borovik | 42.567154 | 18.530277 | 250 |
| *Gentianella crispata* | BIH | Vlasik - Devecani | 44.276779 | 17.626775 | 1000 |
| *Gentianella crispata* | BIH | Pale - Jahorina | 43.715964 | 18.574148 | 1000 |
| *Gentianella crispata* | BIH | Velika Vlahina | 43.730617 | 18.210883 | 250 |
| *Gentianella crispata* | GRC | Kajmakčalan | 40.931111 | 21.789167 | 500 |
| *Gentianella crispata* | XKX | Koritnik | 42.086636 | 20.569095 | 500 |
| *Gentianella crispata* | MKD | turcin sar planina | 41.983619 | 20.797268 | 500 |
| *Gentianella crispata* | XKX | konjuska - sar planina | 42.135735 | 20.939125 | 500 |
| *Gentianella crispata* | BIH | Rujište BIH | 43.449822 | 17.973661 | 500 |
| *Gentianella crispata* | BIH | Visočica | 43.579796 | 18.189883 | 500 |
| *Gentianella crispata* | HRV | Biokovo | 43.341019 | 17.053272 | 500 |
| *Gentianella crispata* | ALB | Çuka e Frëngut and Mount Faqekuq | 40.52784 | 20.41099 | 20 |
| *Gentianella crispata* | ALB | 500 m west of Pass "qafa e Veshit" (2083 m) | 40.56641 | 20.43967 | 20 |
| *Gentianella crispata* | ALB | Mount "Kuq" (2157 m), c. 3.6 km west-northwest of village Shalës and c. 4.6 km east of village Shënepremte | 40.76404 | 20.45302 | 20 |
| *Gentianella crispata* | ALB | "Fusha e Valamarës" | 40.77763 | 20.46749 | 20 |
| *Gentianella crispata* | ALB | eastern slope of Mount Valamarë (2372.6 m) | 40.79281 | 20.47163 | 20 |
| *Gentianella crispata* | ALB | northern slope of Mount "Varri i Pjajakes" | 40.81828 | 20.45898 | 20 |
| *Gentianella crispata* | ALB | Albán-Alpok, Ropojani-völgy, pásztorszállás, sziklahasadékból | 42.45743 | 19.76112 | 20 |
| *Gentianella crispata* | ALB | western slope of Mount "Mëcekut" (1826.4 m) | 41.3753 | 20.02589 | 20 |
| *Gentianella crispata* | ALB | Mali i Dejës, above village Macukull | 41.68859 | 20.1568 | 20 |
| *Gentianella crispata* | ALB | Mali i Dejës, above village Macukull, near pass "Shkol-Den" | 41.68542 | 20.16872 | 20 |
| *Gentianella crispata* | ALB | on the western slope of Mali i Munellës | 41.96623 | 20.09175 | 20 |
| *Gentianella crispata* | ALB | above homestead Gjarpnit a Poshtme, above village Dragobi | 42.46179 | 19.99073 | 20 |
| *Gentianella crispata* | ALB | on the ridge between peaks Maja e Mridzit (1952.3 m) and Maja e Berizhdolit | 42.50666 | 19.71534 | 20 |
| *Gentianella crispata* | ALB | on the slope of mount maja e Trojanit (2194.3 m) | 42.53453 | 19.74209 | 20 |
| *Gentianella crispata* | ALB | on the southern slope of Mt Grykat e Hapëta (2526.4 m) | 42.39681 | 19.9048 | 20 |
| *Gentianella crispata* | ALB | at the foot of Mt maja e Rragamit, above village Kukaj | 42.44745 | 19.84084 | 20 |
| *Gentianella crispata* | MKD | Jablanica - Krstec | 41.232501 | 20.521411 | 500 |
| *Gentianella crispata* | MKD | Crn Kamen | 41.246016 | 20.521848 | 500 |
| *Gentianella crispata* | MKD | Kozuf-Momina Cuka | 41.177633 | 22.203883 | 500 |
| *Gentianella crispata* | MKD | Plackovica | 41.771255 | 22.518826 | 500 |
| *Gentianella crispata* | GRC | Slavjanka (Alibotush) mt.: wet meadows, 1600-1800 m | 41.376031 | 23.620704 | 500 |
| *Gentianella crispata* | MKD | Stogovo mt., Gari village | 41.472945 | 20.679967 | 1000 |
| *Gentianella crispata* | ALB | Shebenikut mt., 1950 m.s.m. | 41.210402 | 20.473972 | 500 |
| *Gentianella crispata* | GRC | Tzena, 5km NNE-N of Notia.... | 41.146665 | 22.215874 | 500 |
| *Gentianella crispata* | MNE | inter pag. Lukovo et Gvozd, Niksic | 42.83767 | 19.052247 | 500 |
| *Gentianella crispata* | BIH | Maglic | 43.275278 | 18.718611 | 50 |
| *Gentianella crispata* | BIH | Dugo Polje | 43.683157 | 17.580789 | 50 |
| *Gentianella crispata* | GRC | Pinovon | 41.10759 | 22.121572 | 1000 |
| *Gentianella crispata* | MNE | Babji Zub | 42.874633 | 19.384894 | 500 |
| *Gentianella crispata* | BIH | Mt. Jadovnik | 44.276827 | 16.415115 | 500 |
| *Gentianella crispata* | MNE | Cakor | 42.692006 | 19.997506 | 250 |
| *Gentianella crispata* | BGR | Golyam Tsarev Vrah | 41.399326 | 23.640436 | 500 |
| *Gentianella crispata* | BIH | Volujak, Herzegovina | 43.231196 | 18.715197 | 500 |
| *Gentianella crispata* | BIH | Bojište | 43.27956 | 18.091743 | 500 |
| *Gentianella crispata* | BIH | Raduša planina | 43.871177 | 17.450834 | 500 |
| *Gentianella crispata* | BIH | Mali Prenj | 43.552959 | 17.914869 | 500 |
| *Gentianella crispata* | BIH | Vucia luka [Vučja Luka] Sarajevo | 43.916654 | 18.517423 | 500 |
| *Gentianella crispata* | BIH | Treskavica | 43.595001 | 18.346505 | 500 |
| *Gentianella crispata* | BIH | Dumoš | 43.36217 | 18.46036 | 500 |
| *Gentianella crispata* | BIH | Zec planina | 43.901974 | 17.842953 | 500 |
| *Gentianella crispata* | MNE | Crivoscie [Krivošija, E part of Orjen] | 42.596556 | 18.626945 | 500 |
| *Gentianella crispata* | MNE | Dziebeze [Mount Debeza] | 42.615375 | 19.549422 | 500 |
| *Gentianella crispata* | MNE | [Mali/Veliki] Mojan | 42.635911 | 19.685123 | 500 |
| *Gentianella crispata* | ALB | Guri i Topit | 40.836454 | 20.450651 | 500 |
| *Gentianella crispata* | MNE | Lovćen | 42.3991 | 18.8184 | 500 |
| *Gentianella crispata* | BIH | Blatačko lake | 43.623587 | 18.108055 | 500 |
| *Gentianella crispata* | SRB | Zmajevački potok, Caričina | 43.234336 | 19.860957 | 50 |
| *Gentianella crispata* | XKX | Peklen | 42.685 | 20.244722 | 1000 |
| *Gentianella crispata* | GRC | Olimbos: near Refuge A/ Spilios Agapitos, 2050-2150 m | 40.079999 | 22.372884 | 200 |
| *Gentianella crispata* | GRC | Olimbos: Mousses Polje, near refuge of Giossos Apostolidis, | 40.094722 | 22.361389 | 200 |
| *Gentianella crispata* | MNE | Ad ### sub m. Kom Vasojevicci [Vasojevići]. | 42.6877778 | 19.667222 | 1000 |
| *Gentianella crispata* | BIH | Alpenwiesen auf der Hranicava pl. Bez. Sarajevo. | 43.7055556 | 18.166944 | 1000 |
| *Gentianella crispata* | BIH | Bosnia: In pratis subalpinis montis Bjelasnica, solo calc. | 43.7033333 | 18.2522222 | 500 |
| *Gentianella crispata* | BIH | Bosnie: Vlasic, plateau calcaire; alt. 1600 m. | 44.2952778 | 17.6394444 | 500 |
| *Gentianella crispata* | BIH | Bosnien, Wiesen b.d. Kaserne in Mestrovac [Meštrevac]. 1000 m. | 43.3072222 | 18.9894444 | 500 |
| *Gentianella crispata* | BIH | Bosnien. Trebević bei Sarajevo, Wiese am Nordhang. | 43.7913889 | 18.4816667 | 500 |
| *Gentianella crispata* | BIH | Bosnien: Wiesen bei der Kaserne Mestrovac [Meštrevac]. 1600 m. | 43.3222222 | 19.0163889 | 500 |
| *Gentianella crispata* | MNE | Durmitor: saddle Mala previja S of Mt. Terzin bogaz | 43.1191667 | 19.0622222 | 25 |
| *Gentianella crispata* | BIH | Flora Bosnae. Veliko Polje in m. Igman pr. Sarajevo. | 43.7477778 | 18.2675 | 500 |
| *Gentianella crispata* | BIH | Flora Bosnae: Treskavica Planina: In pratis ad lacum Veliko jezero. | 43.6055556 | 18.3752778 | 500 |
| *Gentianella crispata* | BIH | Flora von Bosnien. Bjelašnica planina (Vlahinja 1900m). Bez. Sarajevo. | 43.7275 | 18.2088889 | 500 |
| *Gentianella crispata* | BIH | Hänge des Prisjeka bei Popovići (Bez. Glamoč). | 44.1538889 | 16.6833333 | 25 |
| *Gentianella crispata* | BIH | Hercegovina: Nevesinje, in monte Mala Velež. | 43.2863889 | 18.0752778 | 500 |
| *Gentianella crispata* | BIH | Ilica [Ujilica]: Gipfel, Felsen gegen Westen. | 44.2863889 | 16.2263889 | 500 |
| *Gentianella crispata* | BIH | Im Hange des Marino brdo (bei Grahovo) an mehreren Stellen. | 44.1866667 | 16.4866667 | 250 |
| *Gentianella crispata* | BIH | In pratis alpinis montis Porim supra cast. Ruiště (prope Mostar). | 43.4894444 | 17.9213889 | 1000 |
| *Gentianella crispata* | MNE | In pratis sub m. Durmitor c. 1400m. | 43.1416667 | 19.0380556 | 1000 |
| *Gentianella crispata* | MNE | Komovi Mts NW slope of Kom Kučki: mountain pasture Katun Ljuban | 42.6955556 | 19.6469444 | 100 |
| *Gentianella crispata* | BIH | Kräuterreiche Stellen auf Velež planina | 43.3283333 | 18.0033333 | 1000 |
| *Gentianella crispata* | MNE | M. Ključ (1950m) prope Kola. [Kolašin]. | 42.8111111 | 19.6325 | 500 |
| *Gentianella crispata* | MNE | Montenegro, Opština Žabljak. Pitomine: Barno jezero lake; fen in lake litorral | 43.1555556 | 19.0916667 | 20 |
| *Gentianella crispata* | BIH | Mt. Velika Vlanja NNE of Blidinje jezero, NE slopes approx. 1.5km SW of the mountain summit. | 43.7047222 | 17.5294444 | 25 |
| *Gentianella crispata* | BIH | Nevesinje: auf Wiesen des Velež. | 43.2688889 | 18.09 | 500 |
| *Gentianella crispata* | BIH | Plaženica bei Bugojno: am Gipfel. 1700-1766 m. | 44.0580556 | 17.2708333 | 500 |
| *Gentianella crispata* | MNE | Sub jugo M. Lisa et Bar, distr. Vasojevići. | 42.7688889 | 19.6838889 | 500 |
| *Gentianella crispata* | MNE | Süd-Dalmatien: Orjen. | 42.575 | 18.5788889 | 1000 |
| *Gentianella crispata* | BIH | Südgipfel des Jedovnik bei Grahovo [Bosansko Grahovo] im Wasen. | 44.2863889 | 16.3958333 | 500 |
| *Gentianella crispata* | MKD | Tetovo ,Sar Planina, trail from Popova Shapka to Mt. Ceripašina | 42.0144444 | 21.8516667 | 500 |
| *Gentianella crispata* | BIH | Velika Klekovača: Gipfelregion gegen Südosten. | 44.4313889 | 16.5072222 | 500 |
| *Gentianella crispata* | BIH | über die ganze Hochfläche zwischen dem Krstac und dem Ločike (2107m) | 43.9463889 | 17.7425 | 500 |
| *Gentianella crispata* | BIH | eastern slope of Mount Valamarë (2372.6 m) | 43.9261111 | 17.8497222 | 100 |
| *Gentianella crispata* | BIH | Wiesen im Cardak livade [Čardak Livade]: Kalk. ca. 1100 m. | 44.1802778 | 16.9227778 | 500 |
| *Gentianella crispata* | MKD | North Macedonia, Jablanica (Јабланица). Mt. Crn Kamen | 41.241544 | 20.523997 | 10 |

| **Sources:** |  |
| --- | --- |
| **herbaria** | BEOU, LI, LJS, MKNH, NHMR, P, SO, WU, UPA, ZA, ZAHO |
| **pers. comm.** | Zoltán Barina (Albania), Kit Tan (Greece), Milan Veljić (Serbia) |
| **online databases** | Global Biodiversity Information Facility (https://www.gbif.org/), Flora Croatica Database (https://hirc.botanic.hr/fcd/) |
| **literature** | Aneva I, Zhelev P, Evstatieva L, Dimitrov D. 2013. The ecological and floristic characteristics of populations of *Sideritis scardica* Griseb. in Slavyanka Mountain. Bulg J Agric Sci. 19(2):211–217. |
|  | Balijagić J, Markišic H, Jovančević M, Prascevic M, Vincek D, Menković N. 2011. Contribution to the research of medicinal and other plants in the mountainous region of Čakor, Montenegro. Agric For. 57(3):67–91. |
|  | Beck von Mannagetta GE. 1898. Flora von Südbosnien und der angrenzenden Hercegovina. XI. Theil. Ann Nat Mus Wien 13:1–32. |
|  | Bucalo V, Stupar V, Milanović Đ. 2012. Karakteristike i porijeklo populacije molike (*Pinus peuce* Griseb.) na Jadovniku u zapadnoj Bosni. Glas Šumar Banjoj Luci 16:7–29. |
|  | Fiala F. 1899. Beiträge zur Flora Bosniens und der Hercegovina. Wiss Mitt Bosnien Herzegowina 6:719–743. |
|  | Markgraf F. 1931. Pflanzen aus Albanien. Denkschr Akad Wiss. Mat Nat Kl. 102:317–360. |
|  | Millaku F, Rexhepi F, Krasniqi E, Pajazitaj Q, Mala X, Berisha N. 2013. Libri i Kuq i Florës Vaskulare të Republikës së Kosovës. Prishtina: Ministria e Mjedisit dhe Planifikimit Hapësinor të Kosovës (MMPH). |
|  | Hartvig P. 1991. *Gentianella* Moench. In: Strid A, Tan K, editors. Mountain flora of Greece. Vol. 2. Edinburgh: Edinburgh University Press; p. 7–9. |
|  | Pantocsek J. 1874. Phytographische Mittheilungen. Österr Bot Z. 24:140–142. |
|  | Rohlena J. 1931. Achter Beitrag zur Flora von Montenegro. Vestn Král Čes Spol Nauk 35:1–29. |
|  | Sagorski EA. 1912. Neue Beiträge zur illyrischen Flora. Allg bot Z Syst Flor Pflanzengeogr. 18 (1–3):10–18. |
|  | Stadlmann J. 1912. Eine botanische Reise nach Südwest-Bosnien und in die nördliche Herzegowina. Mitt Nat Ver Univ Wien 10:13–21. |
|  | Stešević D., Berg C. 2015. *Botrychium matricariifolium*, a new fern species for the flora of Montenegro. Acta Bot Croat. 74(1):181–186. |
|  | Veljić M, Marin PD, Krivošej Z, Ljubić B 2006. Vascular flora of the Uvac River Gorge in Serbia. Arch Biol Sci 58(2):125–133. |
|  | Wettstein R 1897. Die europäischen Arten der Gattung *Gentiana* aus der Section *Endotricha* Froel. Denkschr Akad Wiss. Mat Nat Kl. 64:309–382. |
