# Supplementary material for: Biogeography of amphi-adriatic Gentianella crispata (Gentianaceae): a northern refugium and recent trans-adriatic migration
Source: Plant Biosyst. 2021 Jun 1;156(3):754–68. doi: 10.1080/11263504.2021.1918783 (PMC13627213; doi:10.1080/11263504.2021.1918783)
Supplement: Supplementary file 1 [file 44473_2022_15603014_MOESM1_ESM.zip › Table_S2.docx]

**Table S2.** Laboratory protocol for cpDNA sequencing of the *Gentianella crispata* samples.

Approximately 20 ng DNA was suspended in 0.7 µl 1x TAE buffer, 4.4 µl ReadyMix (Sigma-Aldrich, Steinheim, Germany), 4.1 µl water, 0.4 µl BSA (1 mg⁄mL; Promega, Madison, WI) and 0.2 µl of each primer (10 µM). PCR conditions were as: 94 °C for 1 min followed by 10 cycles each consisting of 30 s at 94 °C, 30 s at 47 °C, 1.5 min at 65 °C; 15 cycles each of 30 s at 95 °C, 30 s at 49 °C, 1.5 min at 65 °C and a final elongation step of 10 min at 65°C. The PCR programs were executed on GeneAmp PCR System 9700 thermocyclers (PE Applied Biosystems, Foster City, CA, USA). The products were purified using *E. coli* Exonuclease I and FastAP Thermosensitive Alkaline Phosphatase (Fermentas, St. Leon-Rot, Germany) according to the manufacturer’s instructions (37 °C for 30 min and 85°C for 15 min). Cycle sequencing reactions were performed with 4 µl purified PCR product, 0.8 µl BigDye Terminator (PE Applied Biosystems), 1 µl primer (3.2 µM) and 4.2 µl water and finally cleaned with Sephadex G-50 Fine (GE Healthcare Bio-Sciences, Uppsala, Sweden) before being sequenced on an ABI 3770 DNA Analyzer (PE Applied Biosystems).
