# Supplementary material for: Biogeography of amphi-adriatic Gentianella crispata (Gentianaceae): a northern refugium and recent trans-adriatic migration
Source: Plant Biosyst. 2021 Jun 1;156(3):754–68. doi: 10.1080/11263504.2021.1918783 (PMC13627213; doi:10.1080/11263504.2021.1918783)
Supplement: Supplementary file 1 [file 44473_2022_15603014_MOESM1_ESM.zip › Table_S3.docx]

**Table S3.** *Gentianella crispata* populations investigated with cpDNA sequencing, giving number of investigated individuals, number of haplotypes per population and Genbank accession numbers for each population.

| **sampled populations** | | **sequence-derived parameters** | | | |
| --- | --- | --- | --- | --- | --- |
| **population** | **taxon** | **individuals investigated** | **number of haplotypes** | **Accession number rps16-trnQ** | **Accession number rpl32-trnL** |
| **1** | *G. crispata* | 8 | 1 | MN370589-MN370596 | MN370724-MN370731 |
| **2** | *G. crispata* | 7 | 1 | MN370597-MN370604 | MN370732-MN370739 |
| **3** | *G. crispata* | 8 | 1 | MN370605-MN370612 | MN370740-MN370747 |
| **4** | *G. crispata* | 8 | 1 | MN370613-MN370620 | MN370748-MN370755 |
| **5** | *G. crispata* | 8 | 1 | MN370621-MN370628 | MN370756-MN370763 |
| **6** | *G. crispata* | 8 | 2 | MN370637-MN370644 | MN370772-MN370779 |
| **7** | *G. crispata* | 8 | 2 | MN370629-MN370636 | MN370764-MN370771 |
| **8** | *G. crispata* | 7 | 2 | MN370692-MN370699 | MN370823-MN370830 |
| **9** | *G. crispata* | 6 | 3 | MN370645-MN370652 | MN370780-MN370787 |
| **10** | *G. crispata* | 7 | 2 | MN370653-MN370659 | MN370788-MN370794 |
| **11** | *G. crispata* | 7 | 3 | MN370660-MN370667 | MN370795-MN370801 |
| **12** | *G. crispata* | 8 | 5 | MN370700-MN370707 | MN370831-MN370838 |
| **13** | *G. crispata* | 8 | 1 | MN370676-MN370683 | MN370809-MN370816 |
| **14** | *G. crispata* | 7 | 2 | MN370668-MN370675 | MN370802-MN370808 |
| **15** | *G. crispata* | 8 | 2 | MN370708-MN370715 | MN370839-MN370846 |
| **16** | *G. anisodonta* | 8 | 4 | MN370581-MN370588 | MN370716-MN370723 |
