# Supplementary material for: Biogeography of amphi-adriatic Gentianella crispata (Gentianaceae): a northern refugium and recent trans-adriatic migration
Source: Plant Biosyst. 2021 Jun 1;156(3):754–68. doi: 10.1080/11263504.2021.1918783 (PMC13627213; doi:10.1080/11263504.2021.1918783)
Supplement: Supplementary file 1 [file 44473_2022_15603014_MOESM1_ESM.zip › Table_S4.docx]

**Table S4.** Results of the SAMOVA simulations based on cpDNA sequences of *Gentianella crispata* samples. The number of groups was set to K = 2 to K = 14. The fixation indices (FSC, FST, FCT) represents the differentiation between groups, populations in groups and populations, respectively (* p-values <0.05).

| **K** | **FCT** | **FSC** | **FST** | **groups** | **% variation among groups** | **% variation among populations in groups** | **% variation within populations** |
| --- | --- | --- | --- | --- | --- | --- | --- |
| **2** | 0.5295* | 0.7460* | 0.8805* | [1, 2, 3, 4] [5, 6, 7, 8, 9, 10, 11, 12, 13, 14, 15] | 53.0 | 35.1 | 11.9 |
| **3** | 0.5582* | 0.6993* | 0.8671* | [1, 2, 3, 4] [5, 6, 7, 8, 9, 10, 11, 13, 15] [12, 14] | 55.8 | 30.9 | 13.3 |
| **4** | 0.5759* | 0.6544* | 0.8534* | [1, 2, 3, 4] [5, 6, 7, 8, 9, 10, 11, 13] [12, 14], 15 | 62.5 | 23.8 | 13.7 |
| **5** | 0.6730* | 0.5299* | 0.8463* | [1, 2, 4], 3, [5, 8, 9, 12, 13, 14, 15] [6, 7, 11], 10 | 57.6 | 27.8 | 14.7 |
| **6** | 0.7590* | 0.3610* | 0.8460* | [1, 2, 3, 4] [5, 8, 9] [6, 7, 11, 13], 10, [12, 14], 15 | 67.3 | 17.3 | 15.4 |
| **7** | 0.7104* | 0.4523* | 0.8414* | [1, 2, 4], 3, [5, 8, 9, 10, 13] [6, 11], 7, [12, 14], 15 | 75.9 | 8.7 | 15.4 |
| **8** | 0.7904* | 0.2413* | 0.8410* | [1, 2, 4], 3, [5, 8, 9] [6, 7, 11], 10, [12, 14], 13, 15 | 79.0 | 5.1 | 15.9 |
| **9** | 0.7845* | 0.2509 | 0.8386* | [1, 2, 4], 3, 5, [6, 7, 11] [8, 9], 10, [12, 14], 13, 15 | 78.5 | 5.4 | 16.1 |
| **10** | 0.7657* | 0.2995 | 0.8358* | [1, 4], 2, 3, 5, [6, 7, 11] [8, 9], 10, [12, 14], 13, 15 | 76.6 | 7.0 | 16.4 |
| **11** | 0.8056* | 0.1433 | 0.8335* | [1, 4], 2, 3, 5, 7, [6, 11] [8, 9], 10, [12, 14], 13, 15 | 80.6 | 2.8 | 16.7 |
| **12** | 0.8414* | -0.0488 | 0.8337* | [1, 2, 4], 3, 5, 6, 7, [8, 9], 10, 11, 12, 13, 14, 15 | 84.1 | -0.8 | 16.6 |
| **13** | 0.8232* | 0.0454 | 0.8312* | [1, 4], 2, 3, 5, 7, [6, 11], 8, 9, 10, 12, 13, 14, 15 | 82.3 | 0.8 | 16.9 |
| **14** | 0.8033* | 0.1325 | 0.8294* | 1, 2, 3, 4, 5, 6, 7, [8, 9], 10, 11, 13, 12, 14, 15 | 80.3 | 2.6 | 17.1 |
