# Supplementary material for: Biogeography of amphi-adriatic Gentianella crispata (Gentianaceae): a northern refugium and recent trans-adriatic migration
Source: Plant Biosyst. 2021 Jun 1;156(3):754–68. doi: 10.1080/11263504.2021.1918783 (PMC13627213; doi:10.1080/11263504.2021.1918783)
Supplement: Supplementary file 1 [file 44473_2022_15603014_MOESM1_ESM.zip › suppl_material_captions.docx]

**Table S1.** Records of *Gentianella crispata* used only for SDM analysis (i.e., not given in Table 1 and Table S2) giving the iso-coded country, locality, coordinates including uncertainty in meter and data source: either herbarium acronym following Thiers (2019), publication or pers. comm.

**Table S2.** Laboratory protocol for cpDNA sequencing of the *Gentianella crispata* samples.

**Table S3.** *Gentianella crispata* populations investigated with cpDNA sequencing, giving number of investigated individuals, number of haplotypes per population and Genbank accession numbers for each population.

**Table S4.** Results of the SAMOVA simulations based on cpDNA sequences of *Gentianella crispata* samples. The number of groups was set to K = 2 to K = 14. The fixation indices (FSC, FST, FCT) represents the differentiation between groups, populations in groups and populations, respectively (* p-values <0.05).

**Figure S1.** Principal Coordinate Analysis (PCoA) based on a Euclidean distance matrix derived from AFLP data of *Gentianella crispata* samples. Loadings of the first and second axis are 15.58 % and 7.30 %, respectively.

**Figure S2.** Bayesian majority rule consensus tree based on concatenated sequences of trnQ^UUG^– rps16 and rpl32 – trnL^UAG^ intergenic spacers of *Gentianella crispata* samples, bold numbers give the populations, small numbers below nodes the Bayesian posterior probabilities.

**Figure S3.** Species distribution models based on current *Gentianella crispata* distribution data, continuous map with gradient from dark green showing suitable to yellow showing less suitable areas: A) recent distribution of suitable areas including known current occurrences of *G. crispata* symbolized by red dots, B) hindcast of suitable areas during LGM, C) forecast of suitable habitats under moderate future climate warming conditions at 2080.
